# Federated Learning in Smart City Sensing: Challenges and Opportunities

**DOI:** 10.3390/s20216230

**Published:** 2020-10-31

**Authors:** Ji Chu Jiang, Burak Kantarci, Sema Oktug, Tolga Soyata

**Affiliations:** 1 School of Electrical Engineering and Computer Science, University of Ottawa, Ottawa, ON K1N 6N5, Canada; jjian057@uottawa.ca; 2 Faculty of Computer and Informatics Engineering, Istanbul Technical University, Maslak, 34469 Istanbul, Turkey; oktug@itu.edu.tr; 3 Whiting School of Engineering, Johns Hopkins University, Baltimore, MD 21218, USA; tolgasoyata@gmail.com

**Keywords:** federated learning, machine learning, smart cities sensing, internet of things, security, privacy

## Abstract

Smart Cities sensing is an emerging paradigm to facilitate the transition into smart city services. The advent of the Internet of Things (IoT) and the widespread use of mobile devices with computing and sensing capabilities has motivated applications that require data acquisition at a societal scale. These valuable data can be leveraged to train advanced Artificial Intelligence (AI) models that serve various smart services that benefit society in all aspects. Despite their effectiveness, legacy data acquisition models backed with centralized Machine Learning models entail security and privacy concerns, and lead to less participation in large-scale sensing and data provision for smart city services. To overcome these challenges, Federated Learning is a novel concept that can serve as a solution to the privacy and security issues encountered within the process of data collection. This survey article presents an overview of smart city sensing and its current challenges followed by the potential of Federated Learning in addressing those challenges. A comprehensive discussion of the state-of-the-art methods for Federated Learning is provided along with an in-depth discussion on the applicability of Federated Learning in smart city sensing; clear insights on open issues, challenges, and opportunities in this field are provided as guidance for the researchers studying this subject matter.

## 1. Introduction

The global population is witnessing rapid annual growth, especially within urban city settings [1]. Maintaining efficient management of a wide span of information and resources is becoming increasingly more difficult amid growing population, electronic devices, and data transmission [2]. These challenges associated with the growth of such services has motivated governments to look for efficient ways to manage the operation of a city with respect to resource allocation and triggered initiatives around the world to have a connected city system where each component leverages the use of connected technology; these components include the following: economy and finance, citizens, governance, transportation (i.e., mobility), sustainability (i.e., environment), and smart living [3,4,5,6].

**Figure 1:**
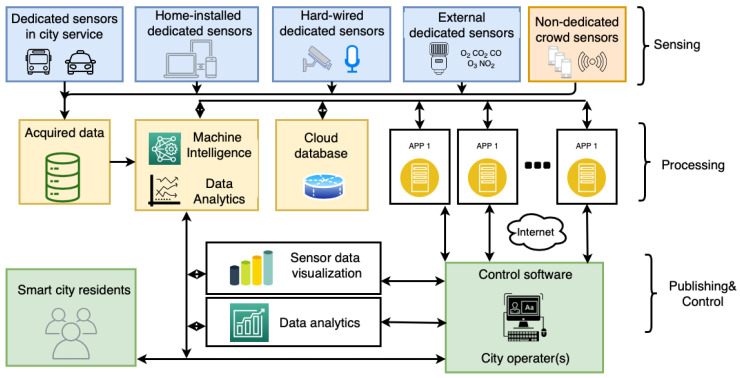
Smart City Sensing Ecosystem [23].

The latest advancements in wireless communication technology have propelled the widespread use of smart technologies, cloud computing, and the Internet of Things (IoT) [7]. IoT is the network of devices that enables connectivity between people, things or services [8,9,10,11]. Advances in manufacturing, sensor and cloud technologies results in a predicted up to 100 billion (with a minimum of 50 billion) devices with Internet connectivity by the end of 2020 [12]. The IoT-cloud environment enables data acquisition and transmission from all parts of a city while the data is processed in the cloud at a centralized server. The widespread use of smart technologies within communities and services has created the building blocks of a smart city [13]. Application areas within smart cities span from smart energy grid, smart transportation services, smart water distribution to smart homes [14,15,16,17,18]. A basic smart city ecosystem is displayed in Figure 1. Sensing as a service is also a vital role that contributes to a smart city [1,19,20]. The Sensing as a Service (S${}^{2}$aaS) concept allows the acquired and aggregated data from embedded/built-in (i.e., non-dedicated) sensors in personal devices available to cloud users. This in turns alleviates companies from the requirement of their own sensing infrastructure. Mobile Crowdsensing is an emerging non-dedicated sensing method within smart cities sensing that uses the falls under the Sensing as a service business model. Where people are recruited into sensing campaigns and are compensated for the data collected by their personal devices [21,22].

Sensing as a service can address many challenges within smart cities [19]. Sensing allows smart services to self-monitor and react to dynamically changing events. An example of this is transportation, monitoring roads, bridges and providing the collected data to more efficiently direct traffic [24]. The data gathered from sensors enables a more efficient resource distribution in a real-time environment. Sensors are becoming increasingly prevalent and abundant within an urban setting, this is due to the reduced production cost of high-quality sensors and the wide spread embedded nature of sensors within smart devices [25]. Various applications can benefit from $S^{2}aaS$ and/or Mobile Crowdsensing so to help propel the big data trend in which large amounts of quality data to become available for processing. The data is often processed with machine learning, deep learning and statistical methods to generate a trend or conclusion [26]. For example, the microphones on mobile devices can be used to monitor noise levels in communities. Sensing as a Service and Mobile Crowdsensing is most prevalent in optimizing traffic control in smart cities. Real-time data relating to traffic congestion, road conditions, parking availability and malfunctioning traffic lights are collected and processed [27]. The best routes can be sent to each individual driver to reduce congestion and avoid accidents. Although known obstacles can be shared as they arise, Brisimi et al. [28] proposed a method of detecting street obstacles that uses smartphone sensing information. This scheme can also be applied to crowd control within a city, such as optimizing the exit route for crowds leaving a sports tournament or concert.

Smart city sensing leverage two main types of paradigms with respect to the operational aspects of sensing systems: (1) dedicated and (2) non-dedicated [23]. Dedicated sensing is when a network of sensors is deployed specifically and permanently for a particular sensing application [27]. There is a plethora of sensors that are distributed throughout a city, however, to reduce the load and expand the sensing capabilities, sensing tasks can be subcontracted to non-dedicated sensors. Although non-dedicated sensing is when mobile sensors are recruited to obtain, process and transmit data to a centralized server, these sensors are not specifically created and deployed for a sensing task, therefore they are considered non-dedicated. Sensor-cloud networks are a novel non-dedicated sensing solution for collaborative sensing tasks in smart cities. A sensor-cloud environment enables the connection of physical sensor nodes to a cloud platform where multiple organization and users can use these sensors for their specific applications [29]. These are considered non-dedicated sensors since the end-user dictates the application and uses of these sensors, while the application changes depending on the need. It is a pay-per-use model that alleviates the massive initial investment cost from one organization [30]. An emerging effective non-dedicated paradigm in smart cities sensing is Mobile Crowdsensing, it is cost effect, highly scalable and has vast mobility. Mobile Crowdsensing allows users participate in a sensing event by providing data through their sensor-enabled mobile devices [31]. Modern phones contain a wide array of sensors such as magnetometer, gyroscope, accelerometer, GPS, camera, proximity sensor, microphone, pedometer, ambient light sensor, barometer and thermometer. The operational differences between the sensing paradigms (i.e., dedicated or non-dedicated) are apparent in several aspects, such as security, sensing performance and implementation cost. Dedicated sensing systems often lead to high expenditures for initial deployment and recurring costs for maintenance, whereas when sensors are not dedicated, it becomes possible to eliminate these upfront costs through using the participant’s pre-existing devices. The challenges for Mobile Crowdsensing is the process of recruiting users and incentivizing users [32,33,34]. The availability of users for a sensing event is not guaranteed due to the irrational ad-hoc nature of Mobile Crowdsensing networks. Furthermore, by using user devices for sensing, the users are exposed to potential privacy leaks [35,36,37,38,39].

This survey covers the capabilities and uses of Federated Learning within smart cities sensing applications. This survey gives a review and a qualitative analysis on how the novel Federated Learning solution can be integrated into smart city sensing to solve the challenges that are currently present. There has been surveys that have extensively covered smart cities sensing [40]. Although Federated Learning is an emerging field, there are a few prominent surveys within this field. The study in [41] covered Federated Learning within mobile edge networks.The authors in [42] presented a survey on the threats to Federated Learning, focusing on poisoning attacks and inference attacks. The authors in [43] presented a survey that detailed the current status and challenges of Federated Learning. This survey compliments the aforementioned surveys by introducing the Federated Learning methodology for smart cities sensing. Smart cities sensing is a key collaborative application that can greatly benefit from the Federated Learning methodology.

The rest of the survey is as follows: Section 2 explains the challenges in current smart cities sensing. Section 3 presents current state-of-the-art Federated Learning solutions. Section 4 shows how Federated Learning can be incorporated into smart cities sensing. Section 5 covers the open issues and challenges remaining in smart cities sensing and Federated Learning methodologies. Section 6 concludes the article.

## 2. Challenges in Smart Cities Sensing

This section covers the major categories that befall smart cities sensing. Habibzadeh et al. [27] classify smart city sensing under dedicated and non-dedicated sensing with the following descriptions for each category: dedicated sensing stands for the traditional way of gathering data where specific sensors are deployed throughout the city to obtain certain sensing data. The range and purpose of these sensors are fixed, therefore optimal planning for node deployment is vital. This limitation also applies to non-dedicated solutions such as sensor-cloud networks. However, within the non-dedicated sensing field, Mobile Crowdsensing that uses sensors present in smart devices to provide sensing services has proven to show comparative advantages with respect to dedicated sensing and sensor networks. Mobile Crowdsensing is becoming more prevalent with its advantages of flexible coverage areas and low overhead costs. The types of data that can be collected is only limited to the sensors present in the smart devices. The data generated can be used to analyze a wider range applications, especially applications related to human behavior since the source of the data is directly from users. This survey explores opportunities and challenges of integrating federating learning with smart cities sensing. However, most nodes deployed for dedicated sensing are currently not equipped with sufficient processing power to support a Federated Learning scenario. Deployment of nodes dedicated sensing is costly due to the initial investment in sensors, equipping these fixed sensors with processors that are capable of training large machine learning models would incur tremendous overhead costs. With the rise in processing power within smart phones and advent of autonomous vehicles, bridging Federated Learning and non-dedicated sensing is a viable solution.

Mobile Crowdsensing is the most common solution to smart cities non-dedicated sensing. With the growth of mobile Internet technology and applications, mobile smart devices have been widely used and greatly popularized [44]. The advent of wireless communication technology and sensor technology makes the use of mobile sensing devices to build a sensing network in a wider range and more complex environment a reality [45]. Smart mobile devices have improved greatly in many areas, such as computing power, storage capacity, and communication capabilities, they also have integrated rich sensors (such as temperature sensors, gravity sensors, acceleration sensors, and so on) and the ubiquitous sensing network make ordinary users able to participate in sensing activities that helps collect the surrounding environmental conditions provides hardware infrastructure support for ubiquitous depth sensing and computing [46]. However, the allocation of huge sensing tasks and the coordination of large-scale sensing devices are the challenges and barriers to achieving ubiquitous depth sensing, as well as computing [47].

Under these circumstances, the proposal and implementation of the Mobile Crowdsensing, which is a cloud-inspired business model, aims at coupling mobile sensing and crowdsourcing towards bridging the gap between hardware infrastructure and ubiquitous depth sensing and computing to form a brand-new IoT sensing model [48], which is a Mobile Crowdsensing network, by coordinating ordinary users’ mobile intelligent devices and mobile sensing devices to perceive their environment and process environmental awareness data through collection, fusion, analysis, mining and other links to restore the user state, situation and environment to collaboratively complete a large sensing task [49]. The mobile network provides a brand-new solution to the complex ubiquitous depth sensing problem [50]. It entails a broad range of scenarios and prospects for applications, and there are new challenges in technology and application research [51].

Mobile Crowdsensing refers to the use of smart mobile devices (e.g., tablets, smart phones, smart wearables, in-vehicle equipment, etc.) that belong to recruited users for a specific sensing campaign and mobile sensing devices as basic sensing units, through mobile Internet or wireless network for conscious or unconscious cooperation to realize the distribution of sensing tasks and the acquisition and processing of the crowd-sensed data to complete complex sensing tasks in real time [52].

Mobile Crowdsensing is usually composed of two parts: the users and the platform [37]. The users are individuals that are recruited for the particular sensing campaign. They use sensors present on smart mobile devices to provide the desired data. The platform is often comprised of a server with data storage centers. The users interact and communicate through the platform according to predefined rules set by the service provider and are compensated based on their contributions [53].

### 2.1. Data Trustworthiness

An efficient Mobile Crowdsensing campaign tightly depends on the truthfulness of the crowd-sensed data. The reliability of gathered data can heavily impact the analysis outcome [54]. This is often done by malicious users or attackers that want to sway the outcome into a scenario that is beneficial to them. This can be done by either submitting false data or by distorting the data during the transmission process. Therefore, ensuring data trustworthiness is a vital step to an efficient and reliable sensing campaign [55]. Cryptographic technologies such as digital signatures, message authentication codes and biometrics are the main methodologies to authenticate the users [56,57,58]. However, these methods do not always guarantee that the data provided by the users is authentic. The data division and other processing measures will bring challenges to the authenticity and integrity of the data [59]. In addition, infrastructure can also be deployed in the sensing area as a reference point and eyewitness for sensing data to verify the authenticity of the sensing data submitted by users, but this solution requires additional expensive infrastructure deployment costs [60,61].

### 2.2. User Incentives

In the Mobile Crowdsensing network, ordinary users are chosen to participate and provide sensing data to complete the social sensing tasks [32,62,63]. However, users participating in sensing needs to pay a certain price (such as consumption of resources, disclosure of privacy, etc.) [34]. Without a certain incentive and compensation mechanism, it is difficult to attract a large participant population to actively participate in large-scale social tasks [33]. Many studies have been proposed based on this incentive mechanism, such as using game theory to explore user habits and preferences, and evaluating and improving the relevance of online search engines [64].

Due to the constraints that limit the participation of users and heterogeneity of the crowdsensing environment affecting the quality of the acquired data, the development of Mobile Crowdsensing has been seriously affected [65]. In response to this problem, the Mobile Crowdsensing incentive mechanism adopts appropriate incentives models to encourage and stimulate participants to participate in sensing tasks [66]. Incentive methods yield different results depending on the participant groups.

The research of the Mobile Crowdsensing incentive mechanism not only needs to adopt appropriate incentive methods, but more importantly, through different incentive methods, solve the core problems faced by both the server platform and the participants in maximizing their respective utility, so as to achieve the role of incentives [67]. The main task of the sensing task server is to incentivize more participants under the condition that the payment cost is minimum, or the payment cost is controllable [68]. Both the participation level of the participants needs to be improved, and the sensing data of the participants must be high quality and reliable [69]. Privacy and resource consumption are two major reasons that prevents a capable user from actively participating in a sensing task [70].

Adopting appropriate incentives can achieve certain incentive effects [71]. However, the study of incentive mechanisms is not only a study of incentives, but more importantly, through the use of reasonable incentives and effective key technologies, both the server platform and the participants maximize the core problems faced by each utility to achieve the role of incentives [72]. These core issues are mainly concerned with: participation level, completion quality, payment control, efficiency and energy consumption, privacy and security, and online real-time processing as reported by Khan et al. [73]. These issues will be elaborated below.

(1) Participation level: The incentive mechanism is used for user recruitment into a sensing campaign. An increased participation rate improves the success rate for the sensing campaign [74]. At the same time, preserving participants will result in long-term collaborations that is beneficial to both parties [35]. The server platform not only expects that participants will join the sensing campaigns, it also expects that any joining participant will remain in the crowdsensing system for an extended length of time to provide long-term data sensing for the sensing task [75]. In addition, the feedback effect of the participation level on the incentive mechanism is also a problem worthy of study [76].

(2) Completion quality: The completion of the sensing task is not only dependent on participation rate, it is also necessary to consider the impact of user location, user behavior, and data quality on the quality of task completion [77]. Mobile Crowdsensing tasks are mostly position-sensitive [78]. The user geo-coordinates will impact the overall quality and value of the sensed data [79]. Participants may intentionally report false data due to their inherent selfishness to affect the perceived data quality [80]. In addition, the sensitivity of the sensing device itself and the limitations of the participants themselves will also affect the task completion quality [81].

(3) Payment control: As a server platform, it is often necessary to pay a certain amount of remuneration to the participants’ perceived data. The payment should be proportional to the quality of user-provided data [82]. Users should be compensated based on the utility they bring to the sensing task [83].

(4) Efficiency and energy consumption: Efficiency and energy consumption not only mean that the server needs an efficient algorithm to process the incentive procedure, but also that the participant hopes that the incentive procedure can lower the resource usage on the sensing device end [84]. The resource consumption of the sensing device is an important factor that prevents participants from participating in a sensing campaign [85]. The mechanism needs to minimize the consumption of these resources [86]. Efficient algorithms are an indispensable part of the incentive mechanism to improve efficiency and reduce running time [87].

(5) Privacy and security: This category includes the privacy concerns of users and the security measures to protect the data in motion and at rest in the server [88]. Participants do not want to disclose personal privacy data when uploading data in sensing tasks, especially location-sensitive Mobile Crowdsensing [89]. Participants may be dishonest Therefore, the uploaded false data poses data security problems for the server [90]. In addition, malicious attacks by malicious users or other entities also need to be considered [91].

(6) Online real time: According to the difference in processing times, the incentives can be processed either online or offline [92]. Offline processing refers to that the server platform needs to make decisions about the allocation of submitted sensing tasks depending on the information gathered from the individuals in a participant pool [93]. The dynamic random participation of participants and the requirement for real-time feedback require an online mechanism to motivate participants in real time [94].

### 2.3. Data Quality Management

The Mobile Crowdsensing network uses the user’s existing equipment for sensing, therefore the sensing data is possibly neither accurate nor reliable [95]. Although it has benefits of low cost, large sensing scale and fine granularity, it also brings a huge challenge in terms of data sensing quality management [96]. If these inaccurate and unreliable sensing data are not processed, it would be difficult to directly use those data in sensing applications [97]. Optimal data quality management is needed for a successful group-aware network. In other words, in a sensing network, data acquisition is easier however quality management for the acquired data is difficult [98]. The key lies in solving the problem of inaccuracy and unreliability of the perceived data, i.e., data sensing quality management. In traditional sensor networks, because sensors can be calibrated before deployment or their sensing tolerances are known, their data sensing quality management is easier, and the key lies in data acquisition, such as how sensors reduce sensing energy consumption, and reliably transfer data to the center [99].

The sources of sensing data are usually different users and sensors, which have the characteristics of multiple modalities and strong correlations. It is necessary to intelligently analyze and explore these data information to find valuable information and make full use of its value, realize the qualitative change from data to information and finally to knowledge [100]. In the exploration of the value of data information, designing the storage and processing of big data, data quality management, and multi-modal data exploration and other aspects of technology is an important challenge [101].

### 2.4. Node Deployment

The complexity and variability of IoT environments present grand challenges for node deployment in the sensing layer. This directly impacts the data collection and analysis process due since the issues will be stemmed from the data source.

Environmental impacts can affect the deterioration of sensor components and thus affect the sensing capabilities of those sensors. Interference might be present in the surrounding areas and cause communication loss. These factors cause unavoidable loss that in turn lead to incomplete data [102].

Another major issue is the coverage area of the deployed nodes. The coverage area is predefined in dedicated sensors; therefore, this metric can be optimized for certain scenarios. It cannot be changed, whereas sensing requirements often change over time. Mobile Crowdsensing sensing nodes are often mobile and can adapt depending on the sensing task. However, the population and device density and participation rate are all factors that can hinder the sensing campaign. Mobile Crowdsensing is completely reliant on the crowd availability. This means that Mobile Crowdsensing sensors performs best in well populated urban metropolitan districts where there is an ample amount of people with smart devices to achieve a wide coverage [103].

### 2.5. Energy Consumption

Resource consumption is still a major challenge in mobile devices during sensing campaigns. These resources include computing, communication and energy resources [104]. Optimizing resource allocation is a key step to improving efficiency in sensing campaigns [105]. However, dynamically allocating resources with for different devices is a difficult task. Oftentimes multiple categories of sensing data are used for one sensing goal, this adds more layers of complexity in the balance of resource allocation. An example of this is when GPS, Wi-Fi and cellular proximity is used to mine positional data. In this case, GPS data consumes the most energy but provides the highest accuracy, a balance between data quality and energy consumption must be evaluated [106].

In the process of continuous application of sensor networks, it is paramount to overcome problems such as node energy, bandwidth and resource constraints in computing, etc., in order to effectively realize its practicality [107]. With the continuous change of time, the quantity of users and the availability of sensors have changed. It is difficult to carry out modeling and prediction work on energy and bandwidth requirements, and it is difficult to complete the sensing task effectively. When selecting an effective subset of users, you need to think about the choice [108]. In the face of a large user pool with different sensing abilities, a more targeted selection methodology needs to be selected. In the case of resource constraints, rationalize the sensing and communication resources [109].

The availability of sensors and sensing tasks changes overtime, therefore developing a model to accurately predict energy consumption with the wide range of unknown and unpredictable parameters is difficult [110].

In addition, many Mobile Crowdsensing applications need to adopt continuous data collection methods to transmit data to the corresponding data centers. The connection between mobile cellular networks and the Internet performs the sensing data transmission, resulting in increased data traffic [111]. Mobile cellular networks bring great pressure. A more effective data transmission method is strongly desired, for example, according to the short-distance wireless communication method, the use of user contact or hotspot sharing to achieve data transmission [112].

### 2.6. User Privacy Protection

Preserving the privacy for participating users in Mobile Crowdsensing campaigns is a top priority for the server. First, a user mobile device can contain sensitive personal data. Secondly, personal information can be concluded by analyzing the data provided by the user. For example, by collecting sensory data related to the user location on the device (such as GPS, electronic compass, magnetic field sensor, etc.), the user’s precise location information can be obtained [36]. Through continued monitoring, the user’s home and work address can be pin pointed and their daily routine can be cataloged [113]. Sensory data can be mined from motion sensors that can be used to deduct the living habits and health information related to a certain user.

Another scenario is when motion sensor data is mined from the user, this information can be used to generate a portfolio of the user’s daily routine and health information. With the combination of environmental sensory data, it would be possible to track and predict the user location at any given time [114]. By collecting the sensory data of the biometric sensor, you can discover the user’s various biometric features, such as sound, images, fingerprints, basic physiological characteristics, and other highly sensitive privacy information. In addition, collecting the daily use data of users can also mine the user’s usage habits, hobbies and behavior characteristics and other deep-level private information [115]. Mobile Crowdsensing enables the optimization of many control sectors, such as pollution, public transportation, traffic congestion, road conditions, etc. However, if sensory data is leaked during the transmission process, it will threaten the privacy of vast amounts of user [116]. Therefore, having appropriate measures to ensure user privacy is preserved is vital in the success of sensing tasks.

## 3. Review of Federated Learning Solutions

In 2016, Google first proposed the concept of Federated Learning, it is a methodology that is often leveraged for joint training of data in multiple edge devices (such as mobile phones) for centralized model training, and is used in scenarios such as input method improvement [117]. As a new generation of artificial intelligence technology, Federated Learning is penetrating the key difficulties of commercial AI application bottlenecks by solving problems such as data privacy and lack of data and it is reshaping the financial, medical, and urban security fields [43].

In modern times, most enterprises have difficulties in obtaining large quantities of quality data for AI model training. At the same time, the regulatory environment is also gradually strengthening data protection, and relevant policies are being introduced continuously [118]. The data owned by commercial companies often have huge potential value. Often these institutions will not provide their own data to other companies, resulting in data often appearing in the form of islands. Mobile phones and wearable devices are very common data generation devices in modern times [119], which generate huge amounts of data in various forms every day. Considering the requirements of computing power, data transmission, and personal privacy, system deployments are increasingly inclined to store data locally, and model calculations are performed by edge devices [120]. The goal is to design a machine learning framework that meets data privacy, security and regulatory requirements so that artificial intelligence systems can leverage the use of their data more efficiently and accurately [121].

Federated Learning is essentially a distributed machine learning technology or machine learning framework. The formal definition of Federated Learning can be defined as: Joint training of machine learning models with distributed devices and local data under federation. Federated Learning requires learning a global statistical model from the massive information stored in millions of remote devices [117]. Federated Learning methodologies enables machine learning models to be trained effectively while ensuring legal compliance by preserving data privacy of the participants. Federated Learning defines a machine learning framework under a virtual model, which is designed to solve the problem of different data owners collaborating without exchanging data [122]. The virtual model is the best model for all parties to aggregate data together, and their respective regions serve local targets according to the model. Federated Learning requires that the modeling result should be infinitely close to the traditional model, i.e., the data of multiple data owners are gathered in one place for modeling results [123]. Under the federated mechanism, each participant has the same identity and status and can establish a shared data strategy. Since the data does not transfer, it does not reveal user privacy or affect data specifications [124].

**Figure 2:**
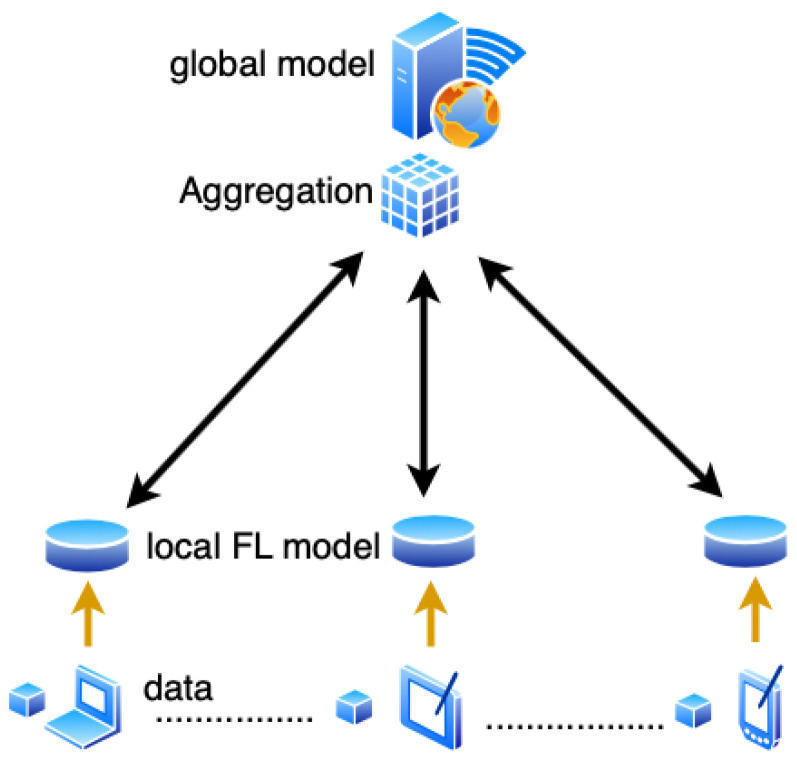
Basic Federated Learning Architecture: Users use local data to train local models, the local models are used to update the global model in the base station. The aggregated global model is passed to the local models for further training. These steps are repeated until the global model converges.

There are three major components of federated learning: data sources, Federated Learning systems, and users. The relationship between the three is shown in the Figure 2. Under the Federated Learning system, each data source performs data preprocessing, then jointly establishes and learns the model, and feeds back the output results to the client. The central server first saves the initial data and distributes it to the participating users [125]. Then the participants uses their own collected local data to train a local model. The parameters of the local model are then transmitted to the central server, while the participating user’s local data remain on their devices [41]. The central server then aggregates the parameters of the uploaded local models to build a global model. The updated global model would then be distributed back to the local users for additional training with local data. This procedure can be re-run until a desired outcome is seen, often when the global model shows a clear convergence. Each user is treated equally without bias in this process [126].

Federated Learning effectively solves the problem of common use of data by two or more data-using entities (clients) without contributing data, and solves the problem of data islands. In addition, under the premise that the data characteristics of each client are aligned, the global model of Federated Learning can obtain the same modeling effect as the centralized storage of data [127].

Federated Learning differs from the traditional distributed machine learning due to the participating devices and dataset properties. In traditional distributed machine learning, the edge nodes are all of equal processing power and the data split is equally divided and often found in Independent and Identically distributed (IID) format whereas in Federated Learning, data is often found in non-IID format, where the data varies in quality, diversity and quantity This is due to the heterogeneous nature of participating hardware devices and variability of using user local data for training [128].

According to the distribution of data sources of the participating parties, Federated Learning can often be split into three types, they are specifically horizontal, vertical and transfer Federated Learning.

Horizontal Federated Learning: When the user characteristics of the two data sets overlap more and the user overlaps less, the dataset is divided horizontally along the user dimension the part of the data that contains the same user characteristics but not the same users is taken out [129].

- Step 1: Each participant downloads the latest model from server A;
- Step 2: Each participant trains the model using local data, uploads the encrypted gradient to server A, and server A aggregates the gradient update model parameters of each user;
- Step 3: Server A returns the updated model to each participant;
- Step 4: Each participant updates their own model.

In traditional machine learning, the data and training are all done in a centralized location and the data is often obtained from data centers. Horizontal Federated Learning can be related to distributed machine learning. Where the difference is that the data used is local device data instead of a distributed partition of data owned by the server. Each machine in Federated Learning obtains the initial global model from the server, training is then done on each device’s local data (followed by the model parameters of the local model) is shared with the server to perform any required updates in the global model. The server aggregates the local model parameters sent by each machine to obtain the global model, the updated global model is then sent back to the participating users and these steps are re-run until a global model convergence is present [130].

In this process, each machine has the same and complete model, and the machines do not communicate and do not depend on each other. During the prediction, each machine can also independently predict. This process can be viewed as a sample-based distributed model training [131]. Google initially adopted the horizontal federated method to solve the issue of locally updating models by end users.

The joint multi-party training methodology in horizontal Federated Learning stems from distributed machine learning. Distributed machine learning represents a distributed split of training data, devices and completion of training [132]. The parameter server is adopted from distributed machine learning, it accelerates the training process by storing data on working nodes while allocating computing resources through the centralized scheduling node [133]. Although the working node becomes the participating users, they have ownership over local data and is independent to the server. Compared to the parameter server where the central node has the highest authority, in Federated Learning the working nodes has the freedom to participate, this adds layers of complexity when it comes to scheduling an optimal learning environment [134]. Horizontal Federated Learning is the strongest candidate for wide adoption in smart cities crowdsensing due to the nature of selective user selection for specific sensing data in Mobile Crowdsensing. Oftentimes, the data would share the same user space and differentiate in feature space. Take smart healthcare as an example: in order to share the same user space, the server would recruit users with the same health illnesses; however, the collected local data would have different features [131].

**Figure 3:**
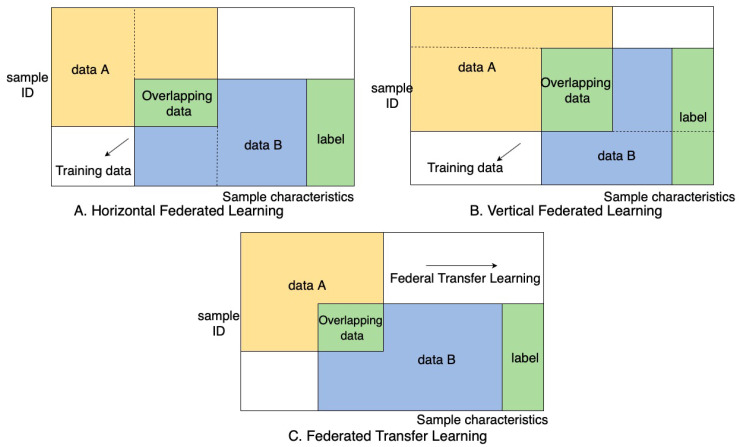
Horizontal, Vertical and Transfer Federated Learning.

Vertical Federated Learning: this is often used when there is an increased user space overlap in datasets and a decreased feature space overlap. The data is then divided in the vertical direction. Vertical Federated Learning allows the aggregation of these split features without interfering with the user privacy requirements [21]. At present, machine learning models are all built under the framework of a vertical Federated Learning system [135]. Examples include but are not limited to logistic regression and decision tree models that are built under the vertical Federated Learning framework. There are two learning steps, which are shown in the Figure 3.

Initially, the encrypted samples are aligned at the system level. This is to ensure that user privacy is not leaked within the enterprise sensing level. The samples are then used for training an encrypted model. The collaborator C sends the public key to A and B to encrypt the data to be transmitted; while A and B train their respective models with their local data, an intermediate step of exchanging gradients and losses occurs between A and B; the gradients are then calculated again after the exchange as well as having an additional mask added, both gradients are then sent to C. C then proceeds to decrypt the received gradients and returns it back to A and B. This is followed by the removal of the mask and updating of the model by A and B [117].

During this process, the participants do not communicate with each other therefore they are unaware of the data features of other participants. After the model is trained, only the portion of the model supported by their own model parameters are returned to them. Since each participant can only obtain the model parameters related to himself, both parties need to cooperate in the prediction. The result of the joint modeling is that both parties obtain data protection and jointly improve the model effect without the loss of model [136].

Vertical Federated Learning can be benefited by the industry or governments more than the masses. A major application for vertical federated learning would be in smart retail or smart finance sector. This is because the data collected from these sectors is often used to profile consumers. For example, the bank may have information relating to consumer spending habits, but a retailer may have information on consumer personal preferences in terms item selection. The bank and retailer cover a large user space that intersect, each providing different features [117].

Federated transfer learning is often used for transfer learning models with deep convolutions neural networks. Using pre-trained models on generalized datasets as a basis and training the scarce amount of data to orient the base model for a specific application. The core of transfer learning is to find the similarity between the source domain and the target domain [137]. The goal of transfer learning is to build effective application-specific models for cases with data is scarce. This is accomplished through leveraging models that are already fully trained and effective for a source domain that is related to the target domain. Then using the available data, to orient the model for use in the target domain. Applications of federated transfer learning would be teaching autonomous vehicles to recognize new signs and road conditions through deep neural networks. Figure 3 shows a representation of horizontal, vertical and federated transfer learning.

A standardized Federated Learning protocol was introduced by Bonawitz et al. [138] to promote the scalability of Federated Learning algorithms. This protocol is repeated during each round of training and consists of 3 steps each time.

- Selection: At the beginning of each training round, a predefined subset of participating users is selected. This selection method can be adjusted or calibrated based on the server requirements or with a custom selection methodology.
- Configuration: The server uses the selected aggregation method and sends the training parameters and model configuration to the selected participants. The participants can proceed to model training.
- Reporting: The participants have trained their models and update the server with their parameters and the server aggregates the updates.

Nishio et al. [139] proposed FedCS, a Federated Learning protocol to improve client selection under heterogeneous device scenarios. The steps of FedCS consists of initialization, resource request, client selection, global model distribution, scheduled update and upload of local model parameters, local model aggregation steps followed by iteration of steps from resource request to local model aggregation. The FedCS protocol enables accelerated federated training process by allowing the server to receive more updates and aggregate more models within the same time frame due to selecting clients based on their resource constraints.

FedGRU was proposed in [140] or small-scale Federated Learning applications, specifically in joint traffic control where private information is often not shared between organizations. The initial global model is pre-trained using public datasets that applies to the selected application domain. The global model is distributed to each participant and trained with local data, and each participant uploads their trained model parameters through encrypted parameters. Finally, the cloud then aggregates all participant models for a new global model followed by distributing it back to each participant.

### 3.1. Aggregation Methods

**Figure 4:**
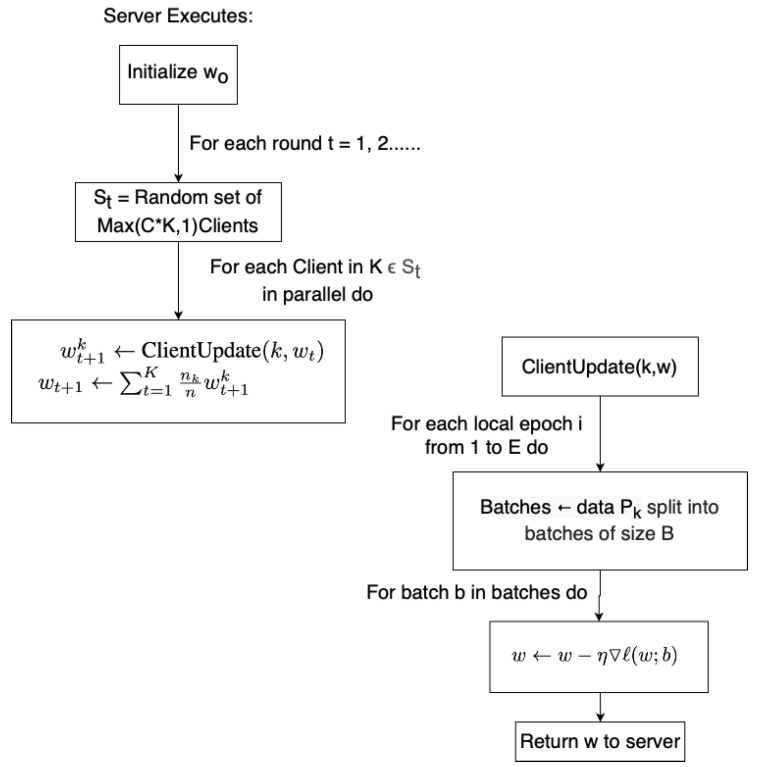
FedAvg algorithm proposed by McMahan et al. [142] that is widely used as a standard algorithm.

The Federated Average (FedAvg) [141] algorithm (illustrated in Figure 4) is an effective yet simple algorithm that is most commonly used for federated aggregation. The FedAvg aggregation consists of equal distribution of model parameters for every local model.

For FedAvg, the gradients of all participants $S_{t}$ is initialized to $w_{o}$. Each round each, local model is trained on its local data and updates the model, given by $w^{t}\leftarrow w^{t}-\eta ▽\ell \left(w;b\right)$ The gradients of the local models are given by $w_{t}$. The gradients are aggregated by the server each round, the updates can be categorized as $w_{t+1}\leftarrow \sum_{k=1}^{K}\frac{n_{k}}{n}w_{t+1}^{k}$ The parameters are averaged between all uploaded models.

The study in [143] propose FedProx to improve upon FedAvg. FedProx tackles the problem of heterogeneity within the Federated Learning environment. This includes the hardware and software variability in participating mobile devices and the statistical heterogeneity by the non-identically distributed data across devices. This is done by introducing a tunable proximal parameter to ensure a better convergence. It addresses statistical heterogeneity by restricting the impact of each local update to the initial global model and addresses system heterogeneity by safely incorporating various degrees of local work.

The study in [144] propose Loss-based Adaptive Boosting (LoAdaBoost) FedAvg to further improve upon FedAvg. This is done so by comparing the loss of the local model in the current epoch to the median loss of the previous epoch. The local model is retrained if it is higher than the previous median loss. A faster convergence is observed with this method, and thus the communication costs can be reduced.

### 3.2. Reputation Models to Ensure Data Trustworthiness

Reputation models have been proposed by [145,146] to ensure reliability and trustworthiness of mobile devices.

The study in [145,147] uses blockchain and multi-weight subjective logic to formulate reputation scores; the reputation is calculated based on previous interactions and opinions of other task publishers. This reputation is then stored within an open-access consortium blockchain. A reputation threshold is set during the user selection, therefore lower reputation users will not be selected. After a model is trained, the performance of the local device is evaluated, using the Reject on Negative Impact (RONI) [148] method for Independent and Identically Distribute (IID) data. As well as using the FoolsGold [149] scheme for non-IID data. RONI detects poisoning attacks by comparing the performance of the local update to a preset update threshold. If the model does not improve over the preset threshold then it will be rejected for global model aggregation. The FoolsGold scheme looks at the gradient diversity of the local updates, if a user repeatedly uploads similar gradients every iteration then it deemed unreliable.

The study in [146] formulates the reputation score of a user by comparing the testing accuracy in three distinct ways. The test accuracy of the local model is compared to the average test accuracy of that epoch, the previous global model test accuracy and the temporary global model test accuracy. A temporary global model is aggregated each epoch to evaluate the capabilities of the combined training of the current epoch, the previous global model is used as a comparison to see how much the local models have improved upon the last epoch. The average test accuracy is used to measure how well the local model is performing compared to its peers. A reputation threshold is set for selecting suitable users to participate in the Federated Learning training. If a user falls below the threshold a set amount of times they will be eliminated from the Federated Learning event.

### 3.3. Privacy Preservation

Privacy Preservation is a key issue in Mobile Crowdsensing, the Federated Learning methodology helps prevent raw data from being sent to the centralized server; however, there are other privacy concerns within the Federated Learning framework and improvements that can be built and incorporated to the Federated framework [150].

Federated Learning methods can leverage Differential Privacy to further prevent information leakage. Oftentimes differential privacy schemes face a challenge to address a trade-off between two objectives: convergence rate and privacy. With this in mind, the authors in [41] propose noising before model aggregation Federated Learning (NbAFL) which satisfies differential privacy by varying protection levels with variances of artificial noise. This method shows that with as the protection level increases the convergence performance decreases, and where an increasing number of clients can improve convergence rate when given a constant protection level.

Liu et al. [150] focused on the improve privacy preservation when sharing model updates without increasing communication cost. They achieved this by proposing sketching algorithms to obfuscate the original data by using independent hash functions. The identities of the user can be concealed during each round of updates due to each user having their own hash indices and seeds.

Hao et al. [151] propose a Privacy Enhanced Federated Learning (PEFL) scheme that uses differential privacy by adding noise according to Gaussian distribution to local models. The perturbed gradients vector of the users is then encrypted into the Brakerski Gentry Vaikuntanathan (BGV) encrypted internal ciphertext. It is then integrated into an augmented learning with error (A-LWE) external ciphertext for secure aggregation. The internal ciphertexts are first all aggregated, then the server decrypts the external ciphertexts. The internal ciphertexts are summed, the aggregated value is easily decrypted by the server while withholding the privacy of the user.

### 3.4. BlockChain

Blockchain is often used to preserve privacy in distributed computing environments. Blockchain has been widely used in Federated Learning schemes.

Awan et al. [152] aim for user privacy preservation, and to do so they propose a method that uses the immutability and decentralized trust properties of blockchain for a secure aggregation process. Their model relies on homomorphic encryption the combined with re-encryption, blockchain and verification. The server generates a pair of private and public task keys while the aggregator generates a pair of private and public batch keys, the public keys are distributed to participating users. The aggregator fuses the updates received from the users, and the aggregated updates are re-encrypted, in which the server would only be able to get the aggregated results. Blockchain also enables easy tracking of client contributions, the contribution can be evaluated by evaluating the global model before and after aggregation.

Lu et al. [153] propose Blockchain, differential privacy with Federated Learning to solve the issue of data islands for industrial IoT applications. For differential privacy, they added noise at the initial stage onto the original data. This may affect the accuracy of the trained models compared to adding noise to the gradients.

Lyu et al. [154] consider fairness in their blockchain-enabled Federated Learning scheme. A local credibility mechanism is used to promote evaluation between users to ensure fairness. Another mechanism to guarantee fairness is that the global server distributes different versions of the global model to the participants based on their contributions. For privacy preservation the authors propose a layered encryption scheme. Blockchain 2.0 is used to store the credibility values of the users, these values are relative to each user’s contributions.

Zhao et al. [155] incorporates blockchain-based Inter-Planetary File System (IPFS) with differential privacy to improve privacy of Federated Learning. Noise is added to the extracted features to ensure differential privacy. Local model updates are sent to the IPFS. IPFS is a file system that allows edge devices to communicate with the same file. Instead of storing actual files on the IPFS, hashes of data location on the blockchain is stored. The immutable nature of blockchains allow for transparency in terms of tracking the model updates from malicious users.

An overview of Research covered within this section can be seen in Table 1.

## 4. Opportunities for Federated Learning in Smart Cities Sensing

Federated Learning can benefit smart cities sensing in multiple aspects [32]. This is most evident when incorporating Federated Learning methodologies for Mobile Crowdsensing tasks, by bridging the gap between data sensing machine learning model training while preserving user privacy. It is possible to use Federated Learning with dedicated smart city sensors; however, oftentimes they do not have the processing power to compute advanced deep learning and machine learning models. The overhead cost of redeploying existing dedicated sensors and manufacturing cost of producing dedicated sensors with higher processing capabilities would be expensive and inefficient. However, in Mobile Crowdsensing the use of personal smart devices that have enough processing capabilities are a prime candidate to integrate with Federated Learning. Along with the growth in smart technologies, more advanced Federated Learning models can be used. This section will go in detail in terms of how Federated Learning can address certain challenges within Mobile Crowdsensing, this includes data privacy, communication costs and training efficiency.

The benefit of the Federated Learning can be seen in two major aspects. First, Federated Learning helps preserve the security of the user by never uploading the raw collected data. Secondly, it is possible to assess the quality of the user’s collected data by testing the local models prior to aggregation.

### 4.1. User Incentives

The additional layers of privacy preservation that is present in Federated Learning schemes will better facilitate user participation.

By having a direct way to measure each individual user’s utility and contribution to the federated sensing campaign, better compensation can be made to the participating users. This will deter users that do not have the capability to provide quality local data.

In Federated Learning, only the trained model parameters need to be uploaded to a server, this represents less bandwidth usage for the user compared to traditional Mobile Crowdsensing. This helps relieve users of high bandwidth costs, which in return incentivizes their participation.

The server in Federated Learning gains a quantifiable revenue from the local data and training progress that the recruited users contribute. Rewarding the participants with viable payout for their contributions is the basis of incentive mechanisms in Federated Learning schemes. The user incentive scheme leverages game theory [117] and contract theory [145]. Games such as the coalition game and labor union game can be used where marginal contribution of the participants are used to gauge pay-offs. Rewards such as reputation, lotteries and auctions can be used as incentive mechanisms. The study in [156] proposes a pay-off sharing incentive scheme that focuses on fairness between participants. Their achieved fairness by modeling the contribution, regret distribution and expectation fairness criteria. Their goal is to concurrently maximize the total utility of the platform by ensuring maximum fairness among participant contributions. The study in [145] proposes a method that uses the junctions of contract theory with reputation scores where higher reputation users would be motivated to users with high-quality data to participate. The authors in [157] propose an incentive method that scores models based on their performance results.

Federated Learning incentive models inherit the advantages of Mobile Crowdsensing incentive methods through the capability of more accurately gauging the utility of a participant. This is due to evaluation methods that can directly measure the performance of their contributed model, evaluating a machine learning model is easier and more concrete than evaluating the contribution of raw sensing data [158].

Federated Learning also allows a user to obtain more payout through the same collected sensing data. Thus, since there will be more computational overhead, a higher payout should be given. Therefore, at the cost of more inconvenience, the users would be able to yield a higher payout by providing the same amount of data. This combined with the security benefits that Federated Learning presents would help incentivize users to participate in Federated Learning-based sensing applications [156].

### 4.2. Data Quality

Mobile Crowdsensing allows users to participate in a sensing event for a particular goal. However, oftentimes, a challenge is to determine the quality of data that each user provides [95]. Integration of Federated Learning-based approaches enables comparison of the loss value or test accuracy of the trained local model to check the viability of the provided user data. The Federated Learning methodology will be able to provide a direct analysis of each user’s data quality. The organizers of the sensing campaign can a predetermined desired output as the model test set and at each epoch the local model performance is tested to see if an improvement is present. If an improvement is seen then the local model is accepted for global aggregation.

This in turn reduces the risk of compensating users that intentionally provide poor data or has device-side issues. This method also directly addresses the device heterogeneity problem within Mobile Crowdsensing, where data is gathered from a multitude of different sensors and devices.

### 4.3. Data Privacy Protection

The ever-increasing stringent Data privacy laws are a key challenge in Mobile Crowdsensing, it is preventing the user recruitment and inhibiting the collection of certain types of sensed data. By never uploading the raw collected data of the users and only uploading the trained local model parameters, the user data is protected can be further protected.

Federated Learning methodologies can incorporate Blockchain and differential privacy to better improve the user privacy [155]. By incorporating noise, the output of the local models will not change the model inherently but will improve the privacy guarantee. The Global model convergence is reduced as privacy is increased with differential privacy. Blockchain is a popular privacy preserving methodology for training neural network models in a distributed environment.

### 4.4. Server Side Overhead

In traditional Mobile Crowdsensing schemes the server must overlook the transmission of data, storage of data and data preprocessing. Mobile Crowdsensing that is used for model training would also need to train the model in a centralized location. These processes incur large overhead costs and maintenance, reducing those requirements would lower the threshold for smart city sensing campaigns.

Mobile Crowdsensing incurs large communication costs with the transfer of raw data into the server. This places a burden on the server itself to process this data and takes a large amount of bandwidth from the users as well. The user resources in terms of bandwidth can be reduced whereas the trade-off is the energy consumption of the devices are increased. In a Federated Learning scheme, the parameter set of the model is transferred to the server as opposed to uploading a bulk of raw data. Lin et al. [159] propose Deep Gradient Compression and it yielded a communication bandwidth reduction in the order of two magnitudes for complex models.

Training complex models is time consuming and requires vast amount of computing power, by leveraging a decentralized approach it will accelerate the training process while providing a more generalized and rich data pool.

### 4.5. Federated Learning Applications

A few examples of Federated Learning applications that contributes to smart city sensing are covered in this section.

#### 4.5.1. Federated Visual Security Sensing

Smart security is an emerging field that is part of the smart cities phenomenon. Traditionally, security relies on the combination cameras, monitoring rooms and personnel to manually detect possible threats. Definitions of abnormalities depend on predefined set of rules. This type of threat detection is labor-intensive and inefficient. Although large amount of data is collected through access cards, cameras, sensors, etc., they are often not used together. The value of the individual data islands is not fully used [160]. Therefore, smart security solutions are highly desired [161].

AI-based model can be used for early warning, real-time high-precision location determination, movement recognition and analysis behavior, to predict travel trajectory and user abnormal behavior, thereby improving community safety and community management efficiency [162]. Federated Learning can be established to train multi-community data for security models while preserving the privacy between communities [163].

Based on machine learning, smart security can perform post event analysis and self-learning, constantly accumulating experience, and continuously improve pre-warning capabilities. Federated Learning offers a machine learning training scheme that allows the use of the large amounts of collected data in daily applications [163].

#### 4.5.2. Federated Autonomous Vehicles

Navigating the dynamically changing, complex and diverse roads is a difficult task for autonomous vehicles [164]. Accidents are highly probable due to the sudden events caused by pedestrians or other vehicles. Industries require large number of drivers for long distance delivery services as a part of the supply chain every year [165]. Autonomous vehicles will improve efficiency by enabling continuous operation without the need for human intervention.

The development of Internet of Vehicles (IoV) and Road Networking technologies will propel autonomous vehicles to become a technological direction with extremely high social value and economic value [166]. Horizontal Federated Learning can be introduced to contribute to more robust machine learning models by fusing sensor information such as cameras, ultrasonic sensors, millimeter wave radar, LiDAR of different vehicles [167]. Vehicular sensing networks are an integral role in smart cities as they provide a vast amount of sensory information and can directly impact smart traffic control [168].

Autonomous vehicles should interact with the IoV, vehicle-road collaboration, and even the entire transportation system to create a better driving environment [169]. Interactive learning should take place between the vehicle and the system environment so that it can assist with other city sensing applications, such as city traffic lights, cameras and road side units through vertical Federated Learning to better integrate information from different sources under privacy protection.

Communication between vehicles is vitally important. Samarakoon et al. [170] propose a Federated Learning-based approach to achieve ultra-reliable low latency communications in vehicles. Lyapunov optimization is used to calculate the joint power and resource allocations to enable low latency communication for vehicular users.

Sensing, decision-making and control are the three core modules of autonomous vehicles. Among them, the sensing of sensor information input such as LiDAR, camera (monocular, binocular, surround-view camera) provides input for the planning stage [171]. At the same time, because the vehicle will generate massive sensory data during the driving process, the original data may involve privacy issues, Federated Learning can be leveraged for real-time adjustments to the AI-based models for dynamic adaptability without compromising the privacy of the data owner.

#### 4.5.3. Federated Aided Diagnosis

Artificial intelligence applications in clinical research are enabled by the digital transformation of medical treatment and clinical information [172]. Medical information is intertwined tightly with patient privacy, and protecting this highly sensitive information is the joint responsibility of all parties including hospitals, artificial intelligence companies and relevant regulatory agencies.

Smart healthcare that leverages Federated Learning can equalize the performance discrepancies between hospitals and provide high-quality test results. By assisting doctors in diagnosis, the burden on the medical system can be reduced [173]. Through standardized data, a horizontal Federated Learning model can be used to ensure that patient data is only kept in the hospital of origin [117]. The Federated Learning model allows for continuous improvements as more medical data is added.

The study in [174] used electronic health records to train an AI model for predicting cardiac events. Smart healthcare based on Federated Learning will empower clinical diagnosis and other subdivisions on the basis of protecting patient privacy, and will promote high-quality medical resources at extremely low cost [175].

## 5. Open Issues, Related Challenges and Opportunities

**Figure 5:**
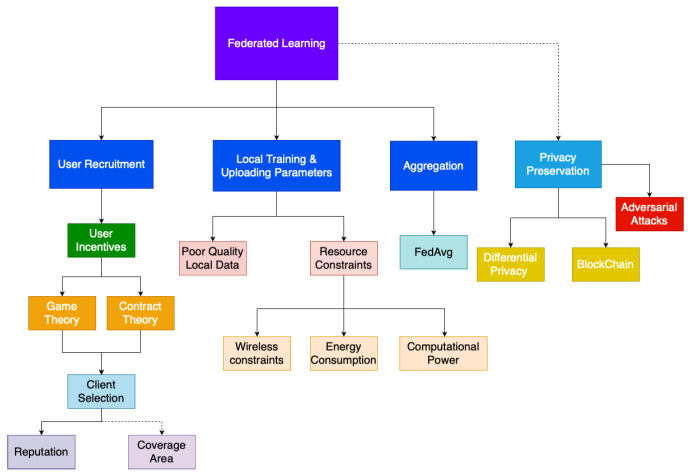
Proposed Taxonomy for Federated Learning.

Figure 5 illustrates our proposed taxonomy of Federated Learning. The major steps in a Federated Learning scheme is user recruitment, local training and uploading of model parameters, aggregation of local models and the privacy preservation mechanics that is used throughout to ensure that the privacy of data is preserved. The blocks highlight in red are the fields that still need further investigation. Coverage area is very crucial in sensing tasks although currently researchers have not focused on the challenges with regards to sensing coverage in Federated Learning schemes. Aggregation methods that can further optimize federated learning performance is still an area that still requires further research, currently most Federated Learning schemes use the FedAvg algorithm for model aggregation; however, application-specific aggregation methodologies can be developed. The energy consumption and heterogenous nature of participating device computational power and their effects on Federated Learning schemes is also an area that offers opportunities for future research.

This section covers the challenges and open issues regarding incorporating Federated Learning into smart city sensing.

### 5.1. Energy Consumption

In Mobile Crowdsensing, energy consumption is a major issue, and it is a metric that is used for calculating the user compensation. By using Federated Learning, the utility of the user can be better gauged; however, Federated Learning consumes much more of the users battery. Depending on the model that is being trained and the amount of local data battery drain can be significant enough to deter users from participating. A proper method to optimize battery usage during Federated Learning is desired, as well as a compensation scheme that is scalable depending on the Federated Learning sensing campaign.

Previous studies have tackled communication challenges regarding Federated Learning [176,177]. However, they often do not take into account the energy consumption of the participating users. Within a fixed training time, unavailability due to outage decreases as energy consumption decreases. Similarly, in the case of a fixed energy consumption guideline, the amount of communication rounds is proportional to outage probability. Therefore, the problem can be oriented by given an energy consumption threshold, optimize the learning performance, or vice versa [178]. The study in [176] optimized energy consumption based on the communication time given to each user as well as the selection of computation parameters. This methodology assumes a strongly convex loss function is present, which is not always the case. The study in [179] optimizes energy consumption as a whole through an adaptive method that gives more bandwidth to users with less computation power and gives priority to participants with strong computation power.

There are many incentive methodologies that have been developed for Mobile Crowdsensing that considers energy consumption. We envision the adaptation of Mobile Crowdsensing incentive methodologies to include the increased energy consumption to appropriately compensate and incentivize participants.

### 5.2. Adversarial Attacks

Federated Learning methodology is susceptible to adversarial attacks, defense mechanisms for a more secure process is still required.

The study in [180] showed how Generative Adversarial networks (GANs) with a multitask discriminator can extract user specific data quietly on the server side. However, the study in [181] showed that adversarial participants can launch white-box membership inference attacks to trace training data records.

Poisoning attacks and inference attacks are most prevalent [42]. Poisoning attacks may target data or local model updates to prevent model training or to initiate a bias towards certain favored features that are beneficial to the adversary. Inference attacks target the privacy of the participants. The exchange of gradients for local updates often can cause privacy leakage [182]. The attacked can conduct property inference by observing the difference between local model updates of a specific user to gain information about that user.

Attacks can be carried out insiders such as server or users as well as outside adversaries. Insider attacks are more impactful than outsider attacks, they are often categorized into a single attack [183,184], byzantine attacks [185] and sybil attacks [149,184].

Participant-level DP can help protect users; however, the exchange in convergence rate and accuracy may not be an attractive solution [142]. Participant-level DP is often used for large participants pools such as thousands of users. Further work is needed to verify the ability of participant-level DP to protect smaller participant pools as well as ensuring that the model converges properly on smaller participant pools [42].

### 5.3. Data Distribution

The data distribution within Federated Learning environment is often categorized into two categories, IID and non-IID data. Non-IID data can be caused by the unbalanced quantity, features and labels.

Non-IID data is much more prevalent situation in a real-world scenario. Zhao et al. [186] showed that non-IID scenarios can lead to significant degradation in the performance of Federated Learning models, which is caused by the weight divergence due to distribution of devices, classes and population. They also suggested a global shared dataset partition to help improve training with non-IID data. They showed that by sharing 5% of data a 30% increase in accuracy can be observed. However, this increases communication costs with models and assumes that such dataset partition is always accessible. This method also increases the susceptibility to data poisoning and adversarial attacks.

Koppararpu et al. [187] propose FedFMC which is a based on a lifelong learning technique [118] for training non-IID data. FedFMC dynamically forks a single global model into different groups depending on its performance on each device dataset. Devices will be grouped to achieve different global models specific to their dataset. This allows the grouped devices to focus on different aspects of the model depending on their dataset properties. At the end, all the forked models are merged. However, this method makes the model more susceptible to an adversarial attack by grouping devices with similar qualities.

## 6. Summary and Conclusions

Federated Learning has appeared as a distributed machine learning concept that uses local data of distributed devices to collaborate with the objective of contributing to the training a global model. It has shown promising performance in preserving the privacy of the participants by training local models and uploading the model parameters to the server instead of uploading the raw data. Privacy preservation is an ongoing challenge in smart cities sensing due to the increasing privacy regulation. A subset of the data gathering from smart cities sensing is often used for training purposes in machine learning models. With these in mind, leveraging Federated Learning for smart cities sensing is envisioned. The participants will be able to provide their data with additional layers of privacy preservation. The quality of data acquired from the participants can be directly gauged by measuring the performance improvements gained due to the contributions of their local data. Communication cost can be reduced with the transmission of model parameters instead of raw data. Moreover, it is possible to empower additional privacy preserving methodologies in Federated Learning-assisted smart cities sensing such as various applications of differential privacy as well as blockchain.

Smart city sensing is an integral part to the smart city ecosystem. This review article has initially presented an overview of smart city sensing and its applications. This has been followed by a discussion on the challenges of smart city sensing in both dedicated and non-dedicated scenarios. These include major areas such as data trustworthiness, user incentives, data quality management, node deployment energy consumption and user privacy protection. By presenting Federated Learning as a promising methodology for preserving privacy within smart cities sensing, state-of-the-art Federated Learning solutions have been presented in Section 3. To present the founding blocks, vertical, horizontal and federated transfer learning as well as other improvements over standard Federated Learning have also been introduced. More specifically in the areas of model aggregation methods, reputation-aware models and privacy preservation methods and Blockchain integration have been reviewed. Lastly, the applications and areas where Federated Learning can help benefit and tackle challenges within smart city sensing have been shown.

Federated Learning is still in its infancy; hence, there are still various challenges faced in adversarial settings. Further research into defense mechanisms is necessary to ensure security of Federated Learning-assisted smart cities sensing. The nature of a Federated Learning environment presents the issue of the statistical heterogeneity of the distributed data, as well as the hardware heterogeneity of participating devices. Although these issues are mitigated through various proposed methods, they have not been eliminated completely. Last but not least, thorough analysis on node deployment and coverage area for federated smart sensing is an issue that needs to be resolved before Federated Learning can be widely adopted for smart cities sensing. 

## Figures and Tables

**Table 1:** Overview of Federated Learning Research covered with key ideas, methodologies, open issues and opportunities for future research.

Area	Ref.	Motivation and Key Idea	Proposed Approach	Open Issues and Further Opportunities
Protocol	[138]	Scalable production system for Federated Learning	Standard protocol as a basis for Federated Learning	Need for optimization for application-specific scenarios
	[139]	Promote client selection under heterogeneous resource scenarios	FedCS protocol to select users based on their resource availability	Relies on the truthfulness of user resource availability submissions.
	[140]	Federated Learning for traffic prediction models	Suitable protocol for small-scale Federated Learning enabled traffic control	Extension to larger scale of recruited clients.
Aggregation	[141]	A standard aggregation method	FedAvg algorithm to aggregate the average model parameters of updates	An alternative to equally weighing all local model updates during aggregation.
	[143]	Optimize Federated Learning in heterogeneous networks	Proximal parameter to limit the impact of variable updates allowing partial work to be done	Solutions for the cases where not all updates are of positive contribution
	[144]	Optimize Federated Learning through data distribution	Loss-based Adaptive Boosting to compare local model losses prior to aggregation	Extensions to consider heterogeneous contribution scenarios during aggregation
Reputation Models	[145]	Incentive to promote reliable Federated Learning	Multi-weight subjective logic to formulate reputation scores	Advanced reputation scores to directly reflect performance of users
	[146]	Enhanced client selection to improve model performance	Local model performance metrics to formulate reputation scores	Minimum computational overhead for assessment of reputation scores for every user
	[147]	Reputation-awareness	Interaction records to generate reputation opinions	Reputation scores to reflect performance of users directly
Differential Privacy	[150]	Enhanced privacy preservation through sketching	Obfuscation of the original data to achieve differential privacy	Performance versus privacy gain
	[41]	Differential privacy in Federated Learning	Noise before model aggregation	Considering varied size and distribution of user data
	[151]	Enhanced privacy and efficiency of Federated Learning in industrial AI applications	Add noise according to Gaussian distribution to local models	Extensive analyses on high-dimensional data
BlockChain	[152]	Accountable Federated Learning	Combine aggregator and blockchain to preserve privacy of users	Fairness assurance in participant rewarding
	[153]	Enhanced privacy for Federated Learning	Noise at the initial stage onto the original data, and use BlockChain to facilitate the Federated Learning process	Tackle potential performance issues due to noising too early
	[154]	Improved fairness and privacy in Federated Learning	Scale rewards with respect to participant contribution	Extension to non-IID scenarios
	[155]	Privacy Preserving Federated Learning for industrial IoT applications	Use blockchain with Inter-Planetary File System (IPFS) and noise to local model features	Extension to non-IID data or heterogeneous device scenarios
